# Neural Network Underlying Recovery from Disowned Bodily States Induced by the Rubber Hand Illusion

**DOI:** 10.1155/2016/8307175

**Published:** 2016-12-27

**Authors:** In-Seon Lee, Younbyoung Chae

**Affiliations:** ^1^Acupuncture & Meridian Science Research Center, College of Korean Medicine, Kyung Hee University, Seoul, Republic of Korea; ^2^fMEG Center, University of Tübingen, Tübingen, Germany; ^3^IMPRS for Cognitive and Systems Neuroscience, University of Tübingen, Tübingen, Germany; ^4^Department of Internal Medicine, Psychosomatic Medicine and Psychotherapy, University of Tübingen, Tübingen, Germany

## Abstract

We used functional magnetic resonance imaging to investigate how causal influences between brain regions during the rubber hand illusion (RHI) are modulated by tactile and visual stimuli. We applied needle rotations during the RHI in two different ways: one was with the real hand (reinstantiation by tactile stimuli, R-TS) and the other was with the rubber hand (reinstantiation by visual stimuli, R-VS). We used dynamic causal modeling to investigate interactions among four relevant brain regions: the ventral premotor cortex (PMv), the intraparietal sulcus (IPS), the secondary somatosensory cortex (SII), and the lateral occipitotemporal cortex (LOC). The tactile aspects of needle rotations changed the effective connectivity by directly influencing activity in the SII, whereas visual aspects of needle rotation changed the effective connectivity by influencing both the SII and the LOC. The endogenous connectivity parameters between the IPS and the PMv were reduced significantly in the R-TS condition. The modulatory parameters between the IPS and the PMv were enhanced significantly in the R-TS condition. The connectivity patterns driven by disowned bodily states could be differentially modulated by tactile and visual afferent inputs. Effective connectivity between the parietal and frontal multimodal areas may play important roles in the reinstantiation of body ownership.

## 1. **Introduction**

The “rubber hand illusion” (RHI) is an experimental paradigm that can manipulate body ownership via congruent touching on the rubber hand and the subject's real hand [1]. The brain interprets the interaction of the visual, tactile, and proprioceptive systems of the body and leads to the recalibration of touch and the felt position of the hand [1, 2]. Several functional magnetic resonance imaging (fMRI) studies have demonstrated that the illusory body ownership during the RHI was highly associated with the parietal and frontal multimodal areas [2, 3] and the lateral occipitotemporal cortex (LOC) [4]. Recently, Limanowski and Blankenburg used dynamic causal modeling (DCM) and revealed the effective connectivity underlying the illusory self-attribution of the rubber hand among four relevant brain regions: the ventral premotor cortex (PMv), the intraparietal sulcus (IPS), the secondary somatosensory cortex (SII), and the LOC [5].

Illusory body ownership during the RHI is known to induce a disowned bodily state for the subject's own hand. Psychologically disrupting the sense of body ownership decreased the awareness of physical self and the physiological regulation of self [6]. Furthermore, illusory ownership over an artificial body part boosted histamine reactivity in the real arm, a key pathway of the innate immune response [7]. Generally, the change in body representation induced by the RHI is considered a temporary phenomenon rather than convincing recalibration of one's bodily representation [8]. However, there has been little interest in investigating how the brain would recover from the disowned bodily state induced by the RHI. Given that models of bodily self-perception are explained by basic spatiotemporal principles of multisensory integration as the key mechanism underlying self-attribution of the body [9], we propose two plausible reinstantiation methods from the disowned bodily states: one involves novel tactile information from the real hand and the other involves novel visual information about the artificial hand.

A stimulating acupuncture needle on the body is known to produce unique sensations and common activation in the sensorimotor cortical network in the brain [10]. In a previous study, we found that participants exhibited reduced, but still prominent, peripheral and central responses to acupuncture needle rotation following the RHI [11]. Furthermore, visual manipulation in the acupuncture stimulation was an important factor for autonomic responses, even without somatosensory tactile stimulation [12]. Taken together, acupuncture stimulation on the body could be a useful tool to reinstantiate body ownership after a disowned bodily state. When needle rotations are provided to the real hand as tactile stimuli, the subject could recover from the disowned bodily state with direct tactile information from his/her own body [11]. In contrast, when needle rotations are provided to the rubber hand as visual stimuli, the subject may recover from the disowned bodily state because visual information from the rubber hand does not correspond to tactile input from the real hand [13]. Thus, it is assumed that the brain networks in the disowned bodily states induced by the RHI could recover in different ways based on two different external information sources.

DCM can provide the strength of effective connectivity and its modulation under experimental conditions between brain regions [14]. We used DCM in conjunction with fMRI to investigate how brain networks are modulated during the RHI by two different stimuli: recovery from the RHI with tactile stimuli to the real hand (reinstantiation by tactile stimuli, R-TS) and recovery from the RHI with visual stimuli to the rubber hand (reinstantiation by visual stimuli, R-VS). Using DCM, we conducted a data-driven estimation of the effective connectivity (causal influence of the activities of certain brain regions on the activities of others), including endogenous connectivity (endogenous connectivity strength independent of experimental condition), and its changes (modulatory effects), under experimental conditions (driving input) between brain regions.

## 2. Methods

### 2.1. Participants

The present study included 17 healthy, right-handed participants (7 females, aged 20–31 years). The participants had no history of neurological, psychiatric, or visual disorders. Each participant received a detailed explanation of the study, and written informed consent was obtained prior to participation. All procedures were performed with the approval of the institutional review board of Korea University, Seoul, Republic of Korea (IRB number KU-IRB-12-48-A-1).

### 2.2. Experimental Design

To induce the RHI, a rubber hand (left hand; Korean Prosthetic Limbs Research Institute, Seoul, Korea) was placed 15 cm above the left hand of the participant. To ensure that the locations of the visual stimuli in the eye-centered coordinates remained the same, the participant was asked to look at the rubber hand throughout the entire experiment, while his/her real hand was completely occluded from view. Details of the experimental design are described in our previous reports [11, 13].

The RHI was induced by gentle strokes with soft brushes. We considered different types of mechanical stimulation, with clear tactile and visual stimulus components that could induce recovery from the illusory state. For example, mechanical stimuli delivering a light sensation, a serious emotional response, such as fear or a threat, and visually ambiguous methods (such as a pad-shape stimulator that could deliver electric or thermal stimuli) were all excluded. Ultimately, rotation of an inserted acupuncture needle was chosen because it provides concise tactile and visual sensory information with no threat. Importantly, all participants had previous experience with acupuncture treatment.

**Figure 1:**
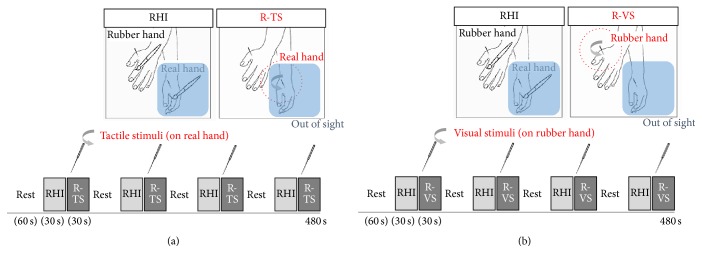
The two sessions involved the same degree of mechanical stimulation (needle rotation) in the real hand (reinstantiation by tactile stimuli or R-TS condition, (a)) and the rubber hand (reinstantiation by visual stimuli or R-VS condition, (b)). Each session included four blocks of brush strokes (30 s, at a frequency of 1 Hz, synchronously on the same location on the rubber and real hand) to induce the rubber hand illusion (RHI) and four subsequent blocks of tactile or visual stimuli (30 s, at a frequency of 1 Hz) immediately thereafter.

Prior to scanning, a needle was inserted at the same location in the real hand and the rubber hand (dorsum of radial to the midpoint of the second metacarpal bone). The two sessions involved an identical degree of mechanical stimulation (needle rotation) in the real hand (tactile stimulus condition) and the rubber hand (visual stimulus condition). All mechanical stimulations were applied by a licensed and experienced doctor of Korean medicine. Each session included four blocks of resting period (60 s), four blocks of stroking brushes (30 s, at a frequency of 1 Hz, synchronously at the same location on the rubber and the real hand) to induce the RHI, and four subsequent blocks of tactile or visual stimuli (30 s, at a frequency of 1 Hz) immediately thereafter. In the R-TS session, the RHI was expected to be modulated by tactile information during needle rotation in the real hand (Figure 1(a)). The participants could not see the stimulation from their real hand. In contrast, the RHI was expected to be modulated by visual information during needle rotation in the rubber hand in the R-VS session (Figure 1(b)).

After fMRI scanning, the participants were asked to assess their perception of the RHI by answering Item 3 on the RHI perception scale: “I felt as if the rubber hand was my hand” [1].

### 2.3. fMRI Data Acquisition

fMRI scans were acquired with a MAGNETOM Trio 3 T scanner (Siemens, Erlangen, Germany) using echo planar imaging (EPI) with a 64 × 64 matrix (TE = 30 ms, TR = 2,000 ms) across 37 slices with a thickness of 4 mm. To minimize movement artifacts, the head of each participant was fixed using a head holder. Each scan session contained 240 volumes of the whole brain in the 37-axial-slice acquisition (TR = 2,000 ms, TE = 30 ms, flip angle = 90°, field of view = 240 × 240 mm^2^, and voxel size = 3.8 × 3.8 × 4.0 mm^3^). As an anatomical reference, a three-dimensional T1-weighted magnetization-prepared rapid gradient echo (MPRAGE) image data set was acquired using the following parameters: TR = 2,000 ms, TE = 2.37 ms, flip angle = 9°, field of view = 240 × 240 mm^2^, voxel size = 0.9 × 0.9 × 1.0 mm^3^, and 192 slices.

### 2.4. fMRI Data Analysis

Preprocessing of the data was conducted using Statistical Parametric Mapping software (SPM8; Wellcome Center for Neuroimaging, London, UK) implemented in Matlab 7.1 (MathWorks Inc., Natick, MA). All participants satisfied a motion threshold of <2 mm spatial displacement in any direction. The data were realigned and coregistered on a mean image, normalized to a template, and smoothed with an 8 mm full-width-at-half-maximum (FWHM) Gaussian kernel. The first four volumes of each session were discarded to allow for T1 equilibration.

For the first-level analysis, a general linear model (GLM) was applied to the preprocessed data. Movements during the scanning sessions were modeled as confounding regressors in the general linear model. RHI and needle rotations were modeled as boxcar functions, convolved with a standard hemodynamic response function that began at the onset of each stimulation, and contrast maps were generated (F-contrast for evaluating the effects of interest, T-contrasts for RHI and needle rotations). A second-level analysis (group analysis) of the stroking brushes during RHI was performed using a random-effects model. This study was performed using the standard summary statistics procedure to make random-effects inferences.

### 2.5. DCM

Our dynamic causal modeling proceeded in two steps. In the first step, we identified the underlying effective connectivity responsible for the rubber hand illusion* per se*. In the second step, we introduced the effects of acupuncture to identify changes in extrinsic coupling or connectivity within the architecture identified in the first step.

In the first step, we explored a number of architectures with bidirectional connections among the four nodes or regions, using the RHI effect as both a driving and a modulatory input. In other words, we modeled the differences between the brain states in the RHI conditions as a mixture of direct driving effects on SII and LOC and a context-sensitive change in coupling between regions. Crucially, we did not differentiate between the two different sorts of reinstantiation (tactile and visual) as RHI procedures before reinstantiation were identical in both sessions, leaving the reinstantiation effects to the second stage.

Having established the best architecture using Bayesian model selection, we then proceeded to the second step. In the second step, we were interested in identifying the regions and connections that differentiated between the tactile and visual reversals of the illusion. We adopted a conservative approach by applying DCM to both sessions separately and then comparing the effective connection strengths using classical statistics (ANOVA) at the between-subject level. This should be contrasted with the more usual approach of modeling both sessions within a single DCM and specifying where the reinstantiation effects could operate (through modulation of endogenous connectivity). We chose the former because it allows for potential effects of reversal on every connection included in the session-specific DCMs. Note that our inferences about the effects of reinstantiation on regional responses and coupling are assessed in relation to between-subject variability using classical statistics. This follows the normal summary statistic approach, in which the estimates from DCM were used to summarize the subject and session-specific neuronal responses. Crucially, we used Bayesian model averaging (within each session) to accommodate the uncertainty about how the reinstantiation effects were mediated. Under the null hypothesis that reinstantiation effects are the same, this session-specific Bayesian model averaging did not introduce any bias into the parameter estimates (i.e., summary statistics).

We performed a standard bilinear, one-state, deterministic DCM using center input using DCM12 implemented in SPM12. Four regions of interest (ROIs) in the right hemisphere (because the stroking brush and needle rotations were delivered to the left hand), the right PMv, the right IPS, the right SII, and the right LOC were selected for three different DCM analyses: (1) the RHI, (2) the R-TS condition, and (3) the R-VS condition. The selected ROIs were reported in a recent paper that provided relevant evidence supporting changes in the effective connectivity between these regions [5].

#### 2.5.1. Definition of the ROIs

The coordinates of the ROIs were based on the aforementioned whole brain GLM (i.e., SPM) analysis and effects of RHI from a previous study (R-TS session: the right SII: 54, −20, 22; the right LOC: 52, −68, 0; the right IPS: 38, −34, 50; and the right PMv: 48, 6, 42; R-VS session: the right SII: 58, −26, 20; the right LOC: 52, −66, 0; the right IPS: 30, −40, 52; and the right PMv: 52, 4, 38) [5]. After the group-level coordinates of each ROI were defined, a 15 mm radius sphere for all ROIs was created and applied as an inclusive mask on individual contrast images for RHI and needle rotations in the R-TS and R-VS sessions (*p* < 0.001, uncorrected). The nearest local maximum to the group-level coordinates within the mask was selected, ensuring that the individual coordinates were within 15 mm from the group coordinates.

The anatomical location of each volume of interest (VOI) was confirmed with neuroanatomical labels from the SPM Anatomy Toolbox [15] and the Talairach Atlas Daemon [16]. The first eigenvariates of all significant voxels within a 6 mm radius sphere centered on individual coordinates were extracted. Because there was no significant activation during needle rotations in one participant in each session, in total, four participants were excluded from the DCM analyses (two in the RHI session, one in the R-TS session, and one in the R-VS session).

#### 2.5.2. DCM for the RHI

As the RHI with stroking brushes was the same in the two sessions, we used all time-series data during the RHI for both sessions for model specification in the DCM analysis during the RHI. In the first step, the endogenous connectivity for the RHI was established, including bidirectional connections between the IPS and the PMv, the IPS and the SII, the IPS and the LOC, and self-connections.

**Figure 2:**
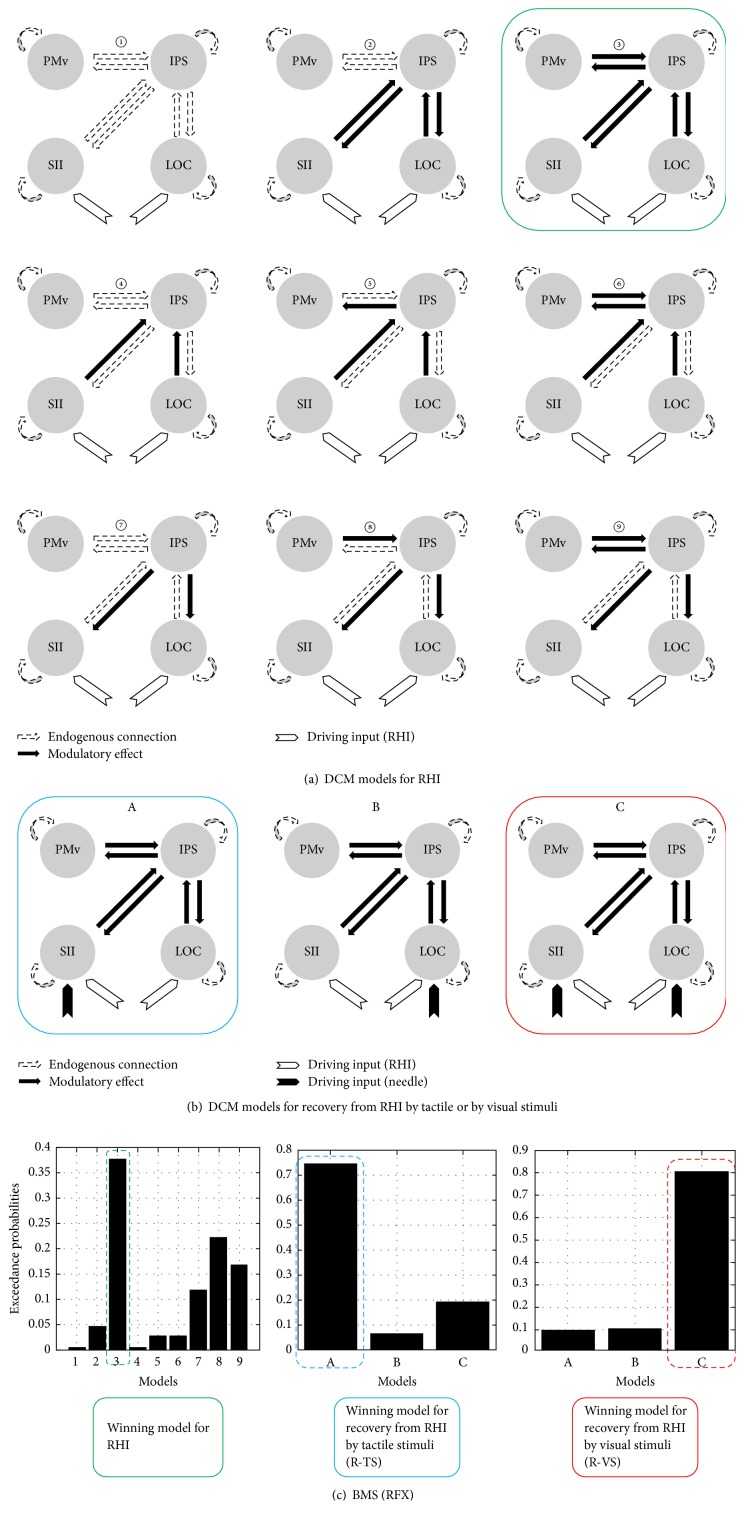
(a) Dynamic causal modeling (DCM) for the RHI (9 models). Blank arrow indicates endogenous connection, and solid arrow indicates modulatory effect. (b) DCM for recovery from the RHI by needle rotation in the real hand (R-TS, 3 models) and in the rubber hand (R-VS, 3 models). Winning models from the random-effects Bayesian model selection for each DCM analysis are marked with a box: Model 3 for the RHI, Model A for recovery from the RHI by tactile stimuli, and Model C for recovery from the RHI by visual stimuli. Winning Model 3 for the RHI was used in DCM analyses for R-TS and R-VS in which the driving inputs from mechanical stimulation were differentially defined as entering into the SII (Model A), into the LOC (Model B), and into the SII and the LOC (Model C). (c) Bayesian model selection (BMS), winning model, and parameter analysis.

The modulatory effect on endogenous connectivity by the RHI was modeled to investigate bottom-up or top-down modulation: no modulatory effect (Model 1), modulation on bidirectional connections between the IPS-SII and the IPS-LOC (Model 2), modulation on bidirectional connections between the IPS-PMv, the IPS-SII, and the IPS-LOC (Model 3), modulation on bottom-up connections from the SII and the LOC to the IPS (Model 4), additional connections from the IPS to the PMv beyond Model 4 (Model 5), additional connections from the PMv to the IPS beyond Model 5 (Model 6), modulation on top-down connections from the IPS to the SII and the LOC (Model 7), additional connections from the PMv to the IPS beyond Model 7 (Model 8), and additional connections from the IPS to the PMv beyond Model 8 (Model 9). Connection parameters were estimated using a Bayesian scheme (Figure 2(a)).

The nine models from both sessions were compared using random-effects (RFX) Bayesian model selection (BMS) after estimation. The winning model, with the highest exceedance probability (Model 3; see Results) was selected as the baseline RHI model for analyses of both the R-TS and the R-VS conditions.

#### 2.5.3. DCM for Recovery from the RHI with Needle Rotation

We evaluated three models for the R-TS and R-VS conditions. To investigate the changes of connectivity strength in the baseline brain network during the R-TS and during the R-VS conditions, the driving input of mechanical stimulation was added to the winning model for the RHI. The models for investigation were thus mechanical stimulation input influencing the activity in the SII (Model A), in the LOC (Model B), and in both the SII and the LOC (Model C) (Figure 2(b)).

We hypothesized that mechanical stimulation could change the brain network of the RHI by directly influencing the activity in the SII (Model A) in the R-TS condition and in both the SII and the LOC (Model C) in the R-VS condition. After estimation, the three models for each session were compared separately using RFX BMS, and the model with the highest exceedance probability was selected as the winning model (Model A for the R-TS condition and Model C for the R-VS condition; see Results).

#### 2.5.4. Bayesian Model Averaging (BMA) and Statistical Analysis (Group Comparison)

As the winning models from the session-specific DCM analyses differed, the parameter estimates from all the models of reinstantiation effects were obtained using Bayesian model averaging (BMA). Exceedance probabilities from BMA analysis of all endogenous connections, modulatory effects, and driving inputs from all participants were extracted and their significance was assessed using a one-sample* t*-test with Bonferroni correction for multiple comparisons. A one-way analysis of variance (ANOVA) was also used to compare the strength of estimated parameters in the three brain networks (RHI, R-TS, and R-VS) with Bonferroni correction.

## 3. Results

BMA was performed across all models to calculate parameter estimates (see Supplementary Table 1 available online at http://dx.doi.org/10.1155/2016/8307175). BMA accounts for individual variability in model fit by weighting parameter estimates by the posterior probability of each model.

### 3.1. The Winning Models

The BMS results showed that Model 3 was the winning model for the RHI (the highest exceedance probability in Model 3 for the RHI = 0.3767). Model A was the winning model for the R-TS condition, whereas Model C was the winning model for the R-VS condition (the highest exceedance probability in Model A for the R-TS condition = 0.7463 and the highest exceedance probability in Model C for the R-VS condition = 0.8053; Figure 2(c)).

### 3.2. BMA Parameter Estimates

#### 3.2.1. DCM for the RHI

Analysis of the parameter estimates of the BMA results for endogenous connectivity showed significant positive connections from the IPS to the PMv (*p* < 0.05), from the IPS to the SII (*p* < 0.001), and from the LOC to the IPS (*p* < 0.01) and self-connections of the IPS (*p* < 0.01) and the SII (*p* < 0.001). In the RHI, the positive connectivity strengths from the IPS to the SII and from the IPS to the LOC were weakened significantly and became negative (*p* < 0.01 and *p* < 0.001, resp.). Driving inputs in the RHI to both the SII and the LOC were also statistically significant (*p* < 0.01 and *p* < 0.001, resp.).

#### 3.2.2. DCM of Recovery from the RHI with Tactile Stimuli

**Figure 3:**
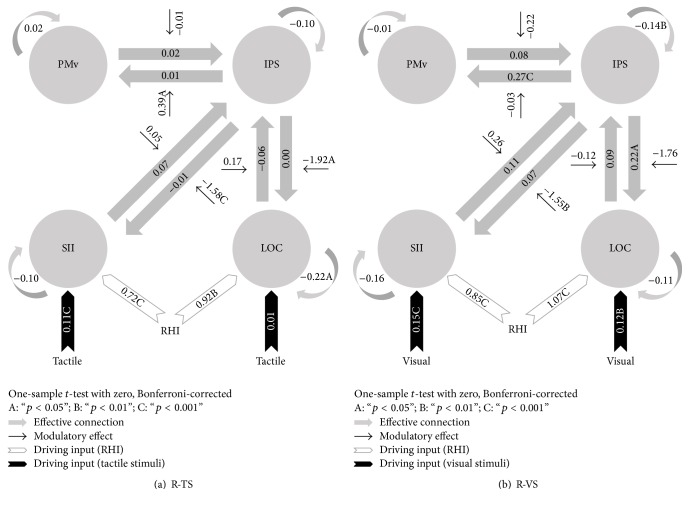
(a) BMA results of recovery from the RHI by needle rotation in the real hand (R-TS condition). (b) BMA results of recovery from the RHI by needle rotation in the rubber hand (R-VS condition). Means of parameter estimates from all participants for endogenous connection (DCM.A), modulatory effect (DCM.B), and driving input (DCM.C) and statistical significance are shown. A one-sample* t*-test with zero was used to assess statistical significance and the Bonferroni correction was applied for multiple comparisons.

Analysis of the parameter estimates of the BMA results for endogenous connectivity showed a significant negative self-connection of the LOC (*p* < 0.05). By needle rotation in the real hand during the RHI, connections from the IPS to the SII and from the IPS to the LOC were weakened significantly and became negative (*p* < 0.001 and *p* < 0.01, resp.) and the positive connection from the IPS to the PMv was enhanced significantly (*p* < 0.05). Only the driving input of mechanical stimulation to the SII was statistically significant (*p* < 0.001; Figure 3(a)).

#### 3.2.3. DCM of Recovery from the RHI by Visual Stimuli

Analysis of the parameter estimates for the BMA results for endogenous connectivity showed significant positive connections from the IPS to the PMv (*p* < 0.001) and from the IPS to the LOC (*p* < 0.05) and a negative connection of self-connections of the IPS (*p* < 0.01). By needle rotations in the rubber hand during RHI, connections from the IPS to the SII were weakened significantly and became negative (*p* < 0.01). Driving inputs in the RHI to both the SII and the LOC were statistically significant (*p* < 0.001 and *p* < 0.01, resp.; Figure 3(b)).

#### 3.2.4. Comparing Effective Connectivity of Recovery from the RHI by Tactile Stimuli

**Figure 4:**
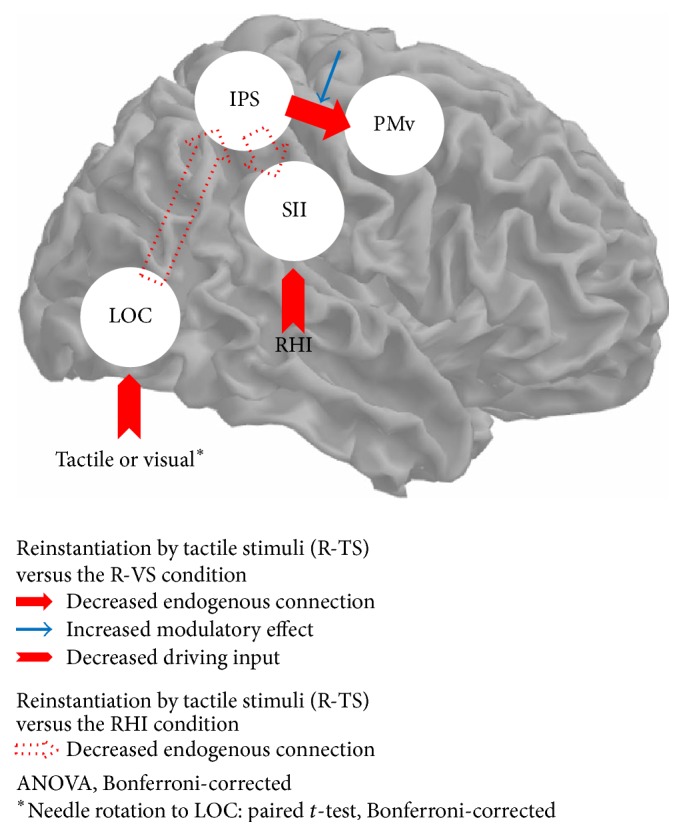
Significant changes in DCM parameter estimates during the R-TS condition versus the RHI (dotted-line arrow) and significant changes in DCM parameter estimates during the R-TS condition versus the R-VS condition (solid-line arrow). One-way analysis of variance (ANOVA) and Bonferroni* post hoc* analyses were used, and the Bonferroni correction was applied for multiple comparisons, except for the driving input of mechanical stimulation to the LOC (*∗*). As mechanical stimulation (tactile or visual stimuli) was modeled in two DCM analyses, paired* t*-tests with a Bonferroni correction were used.

In comparison with the RHI, the endogenous connections from the IPS to the SII and from the LOC to the IPS decreased significantly in the R-TS condition (*p* < 0.05). In comparison with the R-VS condition, the endogenous connection from the IPS to the PMv decreased significantly in the R-TS condition (*p* < 0.05). In comparison with the R-VS condition, the modulatory effect on the connection from the IPS to the PMv increased significantly in the R-TS condition (*p* < 0.01). In comparison with the R-VS condition, the driving input in the RHI to the SII decreased significantly in the R-TS condition, and the driving input of needle rotation to the LOC decreased significantly in the R-TS condition (*p* < 0.05; Figure 4).

## 4. **Discussion**

The present study showed that two different bottom-up information processes with tactile and visual information processing differentially modulated brain networks during RHI-induced disowned bodily states. Tactile information upon mechanical stimulation changed the brain network by directly influencing activity in the SII, whereas visual information on mechanical stimulation changed the brain network by influencing both the SII and the LOC. Importantly, the endogenous connectivity from the IPS to the PMv was reduced significantly in the R-TS versus the R-VS condition. However, the modulatory effect of tactile stimulation was significantly positive in this connection in the R-TS condition, indicating the important role of the connection from the IPS to the PMv in the RHI and in the reinstantiation of body ownership. To our knowledge, this is the first reported study to show the neural network involved in the mechanism underlying the recovery from disowned bodily states induced by the RHI.

In the current study, illusory ownership of a dummy arm was induced successfully by stroking the dummy body part together with the subject's own corresponding body part (0.94 ± 0.32 in R-TS session; 1.17 ± 0.34 in R-VS session) [13]. We first used standard GLM analyses and found that congruent visuotactile touch information following brush strokes selectively resulted in brain activation in the contralateral PMv, IPS, SII, and LOC. These findings were consistent with previous findings in which illusory body ownership was associated with temporoparietal multisensory brain regions [4, 5]. Subsequently, both of the different forms of needle stimulation (tactile and visual) on the body were determined to be effective methods for recovery from the disowned bodily states induced by the RHI. Based on the winning models from the model specification and estimation, the tactile input changed the brain network of illusory body ownership by directly influencing activity in the SII (Model A), whereas the visual input changed the brain network of illusory body ownership by influencing both the SII and the LOC (Model C; Figure 2(b)). Similarly, the two different stimuli differentially changed the activities in the corresponding nodes (driving inputs), leading to changes in the properties of the effective connectivity. The driving input of mechanical stimulation to the SII was significant when participants recovered from the RHI with tactile stimuli (Figure 3(a)). In contrast, the driving input of the RHI to both the SII and the LOC was significant when participants recovered from the RHI with visual stimuli (Figure 3(b)). Although the visual information was delivered from the rubber hand, the brain could still engage with somatosensory input (i.e., the SII) combined with visual input (i.e., the LOC). This can be interpreted as (1) an imagery effect of visual input on other objects or (2) the participants having illusory ownership of the rubber hand. It also supports our previous finding that needle rotations in the rubber hand produced substantial sensation ratings as well as activation in brain areas associated with enhanced bodily awareness of the hand [13]. Furthermore, group comparisons revealed that the driving input of the RHI to the SII decreased significantly in the R-TS session versus that in the R-VS session (red arrow to SII, Figure 4), showing the stronger interference of RHI input to the SII with tactile stimulation. These findings suggest that two different bottom-up sensory information processes attributed to two different external sensory stimuli can result in brain network recovery of disowned bodily states induced by the RHI.

From our DCM results, the connection from the IPS to the PMv could be related to both the formation of illusory body ownership and its reinstantiation. Most accounts of body ownership have been linked to the integration of multimodal information in hierarchical cortical networks, predominantly the PMv and the IPS [17]. The IPS is known to counter prediction error by integrating multisensory touch information and recalibrating the coordinates of the somatosensory reference frame onto the visual reference frame [5]. Increased functional coupling between the IPS and the PMv is known to indicate potential information transfer about the peripersonal space from the parietal cortex to the frontal cortex [18]. The positive enhancement of effective connectivity from the IPS to the PMv by adding new tactile information to the subject's own hand in the R-TS session versus the R-VS session (Figure 4; blue arrow) might result from recalibration of the multimodal peripersonal space into the subject's own body [9]. Taken together, interactions of higher-level integrated brain regions, such as those between the IPS and the PMv, might be involved in the reinstantiation of body ownership from the condition of disowned bodily states. These findings highlight the functional role of connectivity between the parietal and frontal multimodal areas in the reinstantiation of body ownership.

Within the Bayesian theoretical and mathematical framework of the free-energy principle, the brain constantly interprets sensory information by minimizing the average of surprise (i.e., prediction errors) in all the sensory system [19]. Predictive coding suggests that probabilistic representations act as top-down influence on expectations explaining away bottom-up prediction errors between expected and actual sensory events [20]. In the aspect of the principles of free-energy and predictive coding, representations of one's self arise through the integration of sensory information, creating multimodal representations of the self under a hierarchical generative model of the world [21]. The perceptual illusion of body ownership is characterized as inferences of a common cause for visual, tactile, and proprioceptive sensations and modalities, and this can be explained by the Bayesian causal inference [22]. The brain network subserving body ownership is involved in detection of mismatches between the predictions of one's body model and the visuosomatosensory information provided [5]. The influence of ascending somatosensory prediction errors on top-down predictions reduced by the attenuation of somatosensory precision during the RHI [23]. Based on the DCM of electroencephalogram data, perception of the RHI was associated with stronger forward connectivity between visual region and the PMv [24]. In the current study, both needle rotations to rubber hand as visual stimuli and needle rotations to real hand as tactile stimuli could produce another mismatch between expected and actual sensory input, and the brain could reoptimize predictions through the dynamic updating of prior expectations. The reduced endogenous connectivity between the IPS and the PMv in the current study might be associated with the restoration of increased bottom-up influence on the PMv through tactile information of needle stimulation. Our findings suggest that the changed functional architecture of multisensory integration during RHI could be differentially adjusted based on the different external information sources.

In conclusion, this investigation showed that connectivity patterns were differentially modulated for the reinstantiation of the body ownership by adding tactile and visual afferent inputs. Effective connectivity from the IPS to the PMv may be critical to the formation of and recovery from disowned bodily states. Our results thus provide new insight into the underlying neuronal mechanisms for the recovery of body ownership from disowned bodily states.

## Supplementary Material

Table 1: Bayesian model averaging (BMA) results.BMA was performed across all models to calculate parameter estimates. Exceedance probabilities from BMA analysis of all endogenous connections, modulatory effects, and driving inputs from all participants were extracted and their significance was assessed using a one-sample t-test with Bonferroni correction for multiple comparisons. A one-way analysis of variance (ANOVA) was also used to compare the strength of estimated parameters in the three brain networks (RHI, R-TS, and R-VS) with Bonferroni correction. Bayesian model averaging (BMA) results for the RHI (left column, Group 1). BMA results of recovery from the RHI by needle rotation in the real hand (R-TS condition) (middle column, Group 2). BMA results of recovery from the RHI by needle rotation in the rubber hand (R-VS condition) (right column, Group 3).
