# Supplementary material for: Neural Network Underlying Recovery from Disowned Bodily States Induced by the Rubber Hand Illusion
Source: Neural Plast. 2016 Dec 27;2016:8307175. doi: 10.1155/2016/8307175 (PMC5223049; doi:10.1155/2016/8307175)
Supplement: Supplementary file 1 — Table 1: Bayesian model averaging (BMA) results.BMA was performed across all models to calculate parameter estimates. Exceedance probabilities from BMA analysis of all endogenous connections, modulatory effects, and driving inputs from all participants were extracted and their significance was assessed using a one-sample t-test with Bonferroni correction for multiple comparisons. A one-way analysis of variance (ANOVA) was also used to compare the strength of estimated parameters in the three brain networks (RHI, R-TS, and R-VS) with Bonferroni correction. Bayesian model averaging (BMA) results for the RHI (left column, Group 1). BMA results of recovery from the RHI by needle rotation in the real hand (R-TS condition) (middle column, Group 2). BMA results of recovery from the RHI by needle rotation in the rubber hand (R-VS condition) (right column, Group 3). [file 8307175.f1.pdf]

Table 1. **Bayesian model averaging (BMA) results**

		**1. RHI (*n* = 15)**	**2. R-TS (*n* = 16)**	**3. R-VS (*n* = 16)**	**Group comparison**
**Endogenous connectivity**	PMv to PMv	−0.041675 (0.202799)	0.021790 (0.105770)	−0.013673 (0.075633)	ns
	IPS to PMv	**0.177944[a] (0.179579)**	0.005708 (0.284790)	**0.271541[c] (0.173853)**	Group 2 < Group 3*
	PMv to IPS	0.088491 (0.155986216)	0.018664 (0.201168)	0.082392 (0.130200)	ns
	IPS to IPS	**−0.246242[b] (0.184104894)**	−0.102473 (0.125546)	**−0.141254[b] (0.133956)**	ns
	SII to IPS	−0.019684 (0.201039139)	0.074669 (0.183667)	0.113084 (0.295023)	ns
	LOC to IPS	**0.199321[b] (0.17927604)**	−0.061750 (0.175424)	0.091661 (0.276188)	Group 2 < Group 1*
	IPS to SII	**0.275952[c] (0.147039265)**	−0.011409 (0.227519)	0.073475 (0.249834)	Group 2 < Group 1*
	SII to SII	**-0.265858[c] (0.178863548)**	−0.099381 (0.148633)	−0.156575 (0.309578)	ns
	IPS to LOC	0.225554 (0.273540155)	0.002104 (0.405365)	**0.220993[a] (0.266425)**	ns

	LOC to LOC	-0.262745 (0.39078941)	**-0.224111[a] (0.242831)**	−0.109061 (0.236735)	ns
**Modulatory effect in the RHI, R-TS, and R-VS**	IPS to PMv	0.104231 (0.144032)	**0.392094[a] (0.456576)**	−0.030313 (0.264202)	Group 3 < Group 2**
	PMv to IPS	−0.084246 (0.523648)	−0.010238 (0.376813)	−0.222194 (0.808909)	ns
	SII to IPS	−0.221262 (0.475994)	0.053306 (0.841779)	0.257131 (1.100166)	ns
	LOC to IPS	0.221825 (0.588721)	0.168456 (0.512389)	−0.124894 (0.849538)	ns
	IPS to SII	**−0.976345[b] (0.861761)**	**−1.578800[c] (1.117463)**	**−1.548563[b] (1.569129)**	ns
	IPS to LOC	**−1.522450[c] (1.094337)**	**−1.916263[a] (2.061145)**	−1.757388 (2.545317)	ns
**Driving input**	RHI to SII	**0.401545[b] (0.371363)**	**0.719743[c] (0.514789)**	**0.845209[c] (0.436752)**	Group 2 < Group 3*
	RHI to LOC	**0.719957[c] (0.577537)**	**0.923530[b] (0.841429)**	**1.073355[c] (0.788928)**	
	Stimuli to SII	-	**0.109423[c] (0.079169)**	**0.153042[c] (0.118605)**	
	Stimuli to LOC	-	0.006113 (0.064922)	**0.118140[b] (0.111735)**	Group 2 < Group 3 (p < 0.01, paired *t*

Mean (standard deviation; SD). a, b, c: one-sample t-test, Bonferroni-corrected, $p < 0.05$, $p < 0.01$, $p < 0.001$, respectively.

*, **, ***: group comparison, one-way ANOVA, Bonferroni-corrected, $p < 0.05$, $p < 0.01$, $p < 0.001$, respectively.

IPS: intraparietal sulcus; LOC: lateral occipitotemporal cortex; RHI: rubber hand illusion; PMv: ventral premotor cortex; R-TS: reinstantiation by tactile stimuli; R-VS: reinstantiation by visual stimuli; SII: secondary somatosensory cortex.
